# Intake Vaccinations Reduced Signs of Canine Respiratory Disease During an Outbreak at an Animal Shelter

**DOI:** 10.3389/fvets.2021.627580

**Published:** 2021-02-01

**Authors:** Allison Andrukonis, Kelsea M. Brown, Nathaniel J. Hall, Alexandra Protopopova

**Affiliations:** ^1^Department of Animal and Food Sciences, Texas Tech University, Lubbock, TX, United States; ^2^Department of Land and Food Systems, University of British Columbia, Vancouver, BC, Canada

**Keywords:** animal shelter, canine distemper virus, canine infectious respiratory disease, herd immunity, vaccinations

## Abstract

Animal shelters provide an ideal environment for the spread of disease. Dogs are often housed in close quarters with others of unknown vaccine histories, and experience high levels of sustained stress. As a result, Canine Infection Respiratory Disease (CIRD) is often prevalent and difficult to control. The aims of this study were to (1) identify specific pathogens responsible for CIRD in a city shelter in West Texas, USA, and (2) determine whether intake vaccinations decrease proportion of dogs exhibiting signs of CIRD even during an outbreak. A laboratory analysis of conjunctival, pharyngeal, and nasal swabs (*n* = 15 dogs) and fecal samples (*n* = 6 kennels) showed prevalence of various CIRD pathogens (e.g., canine adenovirus-2, canine parainfluenza virus, canine distemper virus). All fifteen dogs tested positive for at least one pathogen, with the most prevalent pathogen being Canine Distemper Virus (CDV; *n* = 12). All of the kennels (n=6) tested positive for Canine Distemper Virus. Health data on dogs (*n* = 1,258) over the age of 6 weeks were assessed from May to August 2017. Beginning in July, both stray and owner-surrendered dogs were vaccinated with Nobivac^®^ Canine 1-DAPPv 5 Way and Nobivac^®^ Intra-Trac^®^ 3 upon intake, which differed from the previous policy. For each day in the study, we calculated the proportion of dogs in each nasal discharge category, the proportion of dogs observed coughing, and the mean fecal score across all dogs. We conducted a linear regression between the proportion of the shelter vaccinated and the proportion of dogs coughing. At the beginning of the vaccination phase, ~25% of the dogs were coughing. However, as the proportion of the dogs vaccinated increased, the proportion of dogs coughing decreased. There was a significant decrease of 7% of the proportion of dogs coughing when vaccination was at least at 90% compared to when it was <90%. These data suggest that the shelter in this study was experiencing a CIRD outbreak, with CDV being primary pathogen, and that it is possible to substantially reduce illness by implementing a vaccination on intake protocol. The current study provides support for the importance of vaccination in animal shelter welfare.

## Introduction

In the United States, it is estimated that 6.5 million companion animals enter animal shelters every year (1). The animal shelter is the ideal environment for the spread of infectious disease: animals are often housed in close proximity to each other with ample opportunities for direct, fomite, and aerosol transmission of pathogens (2, 3). Many are also immunocompromised due to stress, age, and/or current illness (4). Additionally, animals entering shelters may not have received prior vaccinations and are thus completely unprotected from common pathogens (5, 6). Illness at an animal shelter severely reduces the probability of a live outcome. Even when the illness is not severe and may normally be treated easily in the pet home, adopters are less likely to want to adopt a sick animal due to the fear of high veterinary costs, infecting other pets in the family, and even emotional trauma if the new pet passes away (7). Spay and neuter surgeries are also delayed when an animal is showing signs of illness, which may prolong the length of stay at the shelter. Many shelters with fewer resources elect to euthanize companion animals for any signs of illness in order to protect the public, the shelter population, and make space for new healthy animals. As such, the prevention of disease outbreaks in the animal shelter remains the single most important welfare concern for companion animals housed in animal shelters (8).

For dogs housed at an animal shelter, several illnesses are of special concern. Canine Infection Respiratory Disease (CIRD) may be the most challenging to control due to its highly infectious nature (2). CIRD is diagnosed based on clinical signs, such as coughing, sneezing, nasal and ocular discharge, and fever. The responsible pathogens range from those with low mortality such as canine adenovirus-2, canine parainfluenza virus, canine respiratory coronavirus, canine herpesvirus, canine influenza, *Pneumovirinae Paramyxoviridae, Mycoplasma bronchiseptica*, and *Mycoplasma cynos* to pathogens with high mortality such as canine distemper virus (CDV) and *Streptococcus equi* subsp. *zooepidemicus* (2, 9–11). CDV, one of the more challenging pathogens in the shelter environment, is a highly contagious virus of the *Morbillivirus* subfamily of the *Paramyxoviridae* virus that effects a wide range of animals, including the domestic dog (12). Spread through bodily fluids and aerosols, the virus causes a multisystemic infection. Initially replicating in the lymphatic tissues of the respiratory tract, CDV later attacks the gastrointestinal and nervous systems (13, 14). There is no cure for CDV and without proper supportive care, CDV is frequently lethal (2, 15). Mortality rates of CDV are estimated to be around 50% (16). Furthermore, animals that do not succumb to the disease are often left with persistent neurological ticks (15, 17). Correct diagnosis of CDV requires proper testing, frequently through reverse transcriptase polymerase chain reaction (RT PCR). Prevention of CDV as well as other pathogens implicated in CIRD requires vaccination at the point of intake to the shelter (5, 18). However, some low-resourced shelters are still waiting to vaccinate until after the dog has secured an adoption application. This results in a large number of susceptible dogs which oftentimes leads to severe infectious disease outbreaks.

Whereas, the focus of private veterinary medicine is on the health of the individual, shelter veterinary medicine focuses on the health of the entire shelter population. As such, management of infectious disease is considered from the perspective of herd immunity within animal shelters. Herd immunity is the proportion of subjects with immunity in a given population (19), thus representing the health of the overall population. Immunity may be established through prior infection and recovery and immunization. Herd immunity threshold (I_c_) is the point at which the prevalence of protected individuals is higher than the critical proportion. Herd immunity threshold is specific to a microorganism and a population. The simple calculation is as follows:

$$\begin{array}{l} Ic=1-\left(1/Ro\right) \end{array}$$

R_o_ represents the basic reproductive number, which is the mean number of individuals infected by one infectious case during an infectious period. Herd immunity thresholds can also be influenced by a variety factors such as age and demographic settings (20, 21). Herd immunity thresholds are established for many pathogens, such as Measles (22), Polio (22), and more recently Ebola (23).

Whereas herd immunity thresholds are established for many species and populations with varying infectious agents [e.g., in humans (22, 24), amphibians (25, 26), pigs (27), and poultry (28)], it is surprisingly lacking within the context of animal shelter environments. The Association of Shelter Veterinarians mandates intake vaccinations for every animal, with few exceptions. However, herd immunity thresholds for CIRD have not been established. This information is important in order to provide empirically supported guidelines for vaccination requirements in animal shelters.

Dantzler et al. (29) created a theoretical model of CDV spread in an animal shelter. The shelter on which the model was created had a robust vaccination-at-intake model, thus vaccination was held constant. The authors concluded that while many variables affect the spread of CDV, adoption and euthanasia rate are inversely proportional to the spread rate (29). However, they suggested that more information is needed on evaluating vaccination programs and their effect on CDV spread. With additional information about herd immunity thresholds for CDV, and the presentation of CIRD in brick-and-mortar shelters, future improved models may be created. We expect that by incrementally increasing the vaccinated population from ~0 to 100% and simultaneously recording the clinical signs of CIRD, will contribute to the knowledge needed for improved models.

In this study, we aimed to (1) identify specific pathogens responsible for CIRD in a medium-sized urban city shelter in West Texas, USA, and (2) determine the effects of implementing a vaccination program to reduce an outbreak.

## Materials and Methods

### Animals

Dogs of unknown breeding (*n* = 1,258) over the age of 6 weeks and housed at the Lubbock Animal Shelter, a municipal shelter in Lubbock, Texas, were enrolled in the study from May to August 2017. The shelter is an open admission facility, which admits stray, owner-surrendered, and seized animals. For this study, health data and diagnostic swabbing were collected on dogs in the stray ward (28.9 × 15.4 m), and vaccinations were provided to all dogs upon intake during the experimental phase. Only dogs too aggressive to safely handle were excluded from vaccinations (*n* = 8).

Dogs were primarily group housed (range = 1–14 per kennel) in steel kennels (1.6 × 1.2 × 1.9 m) with cement siding and floors. A guillotine door divided the two parts of each kennel. Occasionally the guillotine door was raised, giving the dogs access to both sides of the kennel. Dogs were housed in the stray ward if they entered the shelter without any form of owner identification (e.g., collar tag or microchip) or were owner-surrendered. The front two rows of kennels in the stray ward were for male dogs, the middle two rows of kennels were for female dogs, and the back row was used for pregnant/lactating, injured, or aggressive dogs. On average, there were 163 dogs housed in the stray ward on a daily basis. Staff cleaned the kennels daily at 8:30 am and dogs were fed and given water following the cleaning. Kennels did not include a bed or enrichment items, except for rare situations. Most dogs were never taken out of their kennels.

### Data Collection

#### Health Data

Baseline health data were collected from May 1st, 2017- May 24th, 2017 on Mondays, Wednesdays, and Fridays, at 8 a.m. CT. An experimenter walked by each kennel and recorded the presence of coughing, and nasal discharge for individual dogs. We focused on coughing and nasal discharge because previous research showed that these clinical signs were predictive of fever and subsequent illness for dogs in animal shelters (30). The operational definitions of these categories are listed in Table 1. If multiple types of nasal discharge were observed in a given dog, we documented the most severe, with clear discharge being the least and bloody discharge being the most severe. Dogs were documented and tracked using their unique intake ID number. Health data during the vaccination period were collected in the same manner.

**Table 1:** Operational definitions of health data categories.

**Description**	**Operational Definition**
Coughing	Sudden, noisy, forceful expulsion of air from the lungs while the mouth remains open. May sound similar to human hacking
**Nasal Discharge**	
No discharge	Nose is relatively dry with no crusting
Clear discharge	Nose appears wet, transparent drops of discharge evident
Yellow/green discharge	Opaque and viscous yellow or green colored discharge
Crust on nose	Crusting on and around the nose is present
Crust and discharge	Opaque and viscous yellow or green colored discharge. Crusting on and around the nose is present
Bloody discharge	Blood or red colored discharge is present

An additional experimenter walked by all of the kennels and recorded the presence and score of the feces in each kennel using Purina's Fecal Scoring System. Possible scores ranged from 1, very hard and dry stool, to 7, liquid stool with no texture. Kennels were scored based on the highest fecal score.

On 472 (8%) of observations, two observers independently collected data to calculate interobserver agreement (IOA). IOA was calculated using Cohen's Kappa [(31); see Table 2].

**Table 2:** IOA values.

**Category**	**Percent Agreement**
Coughing	74.3
Nasal Discharge	67.5
Nasal Discharge- absence/presence	73.1
Fecal Score	59.6

#### Pathogen Testing

Dogs (*n* = 15) were tested by researchers using the University of Florida Maddie's^®^ Shelter Medicine Program instructions (32). Swabs were collected from ten dogs with clinical signs of illness (e.g., coughing, nasal discharge, fever), and five that were not displaying clinical signs. For each dog, a conjunctival swab, a deep nasal swab, and a deep pharyngeal swab were collected. The three swabs were placed into the same dry sterile tube. Approximately one-third of each swab stick was placed into the sterile tube and then the ends of the swabs were snapped off. Each tube was labeled with the dog's unique intake ID number. The conjunctival swab was collected by pulling the lower eyelid away from the eyeball, exposing the conjunctival sac. The tip of the swab was then inserted into the sac and firmly rubbed against the inside of the eyelid, removing epithelial cells. Deep pharyngeal swabs were collected by opening the mouth and vigorously swabbing the back of the oropharynx near the tonsils. Deep nasal swabs were collected by inserting the swab tip as far into the nostril as possible and rubbing to remove epithelial cells. Samples were shipped on ice to IDEXX (IDEXX Laboratories, Inc., Westbrook, Maine) for PCR testing, where they ran a Canine Respiratory Disease panel (33).

Fecal pathogens were also evaluated in kennels (*n* = 6). Several samples of feces, both solid and liquid were collected from each pen. Feces from the individual pens were mixed together in a tube. The total amount of feces in each tube was approximately the size of a walnut. The tubes were then labeled with their respective kennel numbers and submitted to IDEXX for the Canine Diarrhea PCR Panel.

#### Vaccinations

Researchers began vaccinating on 7/3/2017 and continued until 8/25/2017. Upon intake, both stray and owner-surrendered animals were vaccinated with Nobivac^®^ Canine 1-DAPPv 5 Way (Merck Animal Health, 1995-2018) and Nobivac^®^ Intra-Trac^®^ 3 (Merck Animal Health, 1995-2018). Nobivac^®^ Canine 1-DAPPv, given subcutaneously, is a 1-year modified-live vaccine that provides protection against canine distemper, adenovirus type 1 (hepatitis), adenovirus type two (respiratory disease), canine parainfluenza virus, and canine parvovirus. Nobivac^®^ Intra-Trac^®^ 3, given intranasally, is a 1-year modified-live and avirulent live culture vaccine that provides protection against adenovirus type two, canine parainfluenza virus, and *Bordetella bronchiseptica* infection.

Only dogs *entering* the shelter were vaccinated; the dogs that resided at the shelter prior to 7/3/2017 (*n* = 143) remained unvaccinated (or unknown, as some may have been vaccinated in the community, for example by previous owners). The reason for this was 2-fold. First, based on the crowded housing environment and continuous rotation of dogs, it was reasonable to assume that all resident dogs had already been exposed to pathogens. Second, by vaccinating on intake we were able to gradually transition from a population in which none of the dogs had documented vaccination records to a population in which nearly every dog present was vaccinated. This allowed us to track the proportion of sick individuals in relation to the proportion of the population that was vaccinated.

Research personnel were trained to vaccinate by a licensed veterinarian and vet tech. Research personnel were present in the animal shelter during open hours. Dogs were vaccinated as soon as they entered the shelter. Owner-surrendered dogs and stray dogs brought in by citizens were vaccinated in the intake room at the shelter. Stray dogs brought in by officers were vaccinated either in the sally port or intake room. Dogs brought in after hours were vaccinated in their kennels at 8 a.m. the next day.

#### Statistical Analysis

Nasal discharge was broken down into three categories: none, clear discharge, and other discharge. Yellow/green discharge, crust on nose, crust and discharge, and bloody discharge were considered other discharge. For each day in the study, we calculated the mean fecal score across all dogs, the proportion of dogs in each nasal discharge category, and the proportion of dogs observed coughing. Proportion coughing was used as a primary dependent measure to evaluate CIRD as this was previously shown to be highly associated with overall health of the animal and rectal temperature (30). Nasal discharge was used to confirm the coughing analysis. To evaluate whether the vaccination rate was associated with the proportion of dogs coughing and with each nasal discharge category, we calculated the proportion of dogs coughing and presence of nasal discharge for each day from the start to end of the vaccination period. The baseline period was excluded due to potential seasonal effects. For each of these days, we calculated the proportion of the dogs who were vaccinated at that point. We then conducted a linear regression between the proportion of the shelter vaccinated and the proportion of dogs coughing. For the days during the vaccination period, we compared the proportion of dogs coughing when the vaccination status was <90% and when the vaccination status was >90%. Finally, we compared the proportion of dogs with each nasal discharge category when the vaccination status was <90% and when the vaccination status was >90%.

## Results

### Pathogen Results

Table 3 shows the results from swabbing 15 dogs for pathogens. All fifteen dogs tested positive for at least one pathogen. Of the 11 pathogens measured, seven were present in at least one dog. The most prevalent pathogen was Canine Distemper Virus, with 80% (*n* = 12) of dogs testing positive. Coronavirus, and Pneumovirus were the next most frequent, both with 46.7% (*n* = 7) of dogs tested positive.

**Table 3:** Results from IDEXX PCR pathogen testing on 15 dogs.

	**Dog ID**															
**Pathogen**	**1**	**2**	**3**	**4**	**5**	**6**	**7**	**8**	**9**	**10**	**11**	**12**	**13**	**14**	**15**	**Percent Positive**
Canine Distemper Virus	–	+	+	–	–	+	+	+	+	+	+	+	+	+	+	80
Canine Respiratory Coronavirus	–	–	–	+	+	+	+	–	+	–	–	+	–	–	+	46
Canine Pneumovirus	–	–	+	–	+	+	+	–	–	–	+	+	–	–	+	46
*Mycoplasma cynos*	+	–	+	+	–	–	–	–	–	–	+	+	–	+	–	40
Canine Adenovirus-2	–	–	–	–	–	–	+	–	+	–	+	–	–	–	+	26.7
*Bordetella*	+	–	–	+	–	–	–	–	–	–	–	–	–	–	–	13.3
Canine Parainfluenza	–	–	+	–	–	–	–	–	–	–	+	–	–	–	–	13.3
H3N2 Influenza	–	–	–	–	–	–	–	–	–	–	–	–	–	–	–	0
Canine Herpesvirus	–	–	–	–	–	–	–	–	–	–	–	–	–	–	–	0
*Influenza A*	–	–	–	–	–	–	–	–	–	–	–	–	–	–	–	0
*Strep Zoo*	–	–	–	–	–	–	–	–	–	–	–	–	–	–	–	0

Table 4 shows the results from the fecal collections of the six randomly selected kennels. Of the 13 pathogens tested, six were present in at least one kennel. Canine Distemper Virus was present in 100% of the kennels tested. Enteric coronavirus was present in 50% (*n* = 3) and *C. perf* alpha Toxin Gene and *C. perf* CPnetEF Toxin Gene were both present in 33.3% (*n* = 2) of the kennels.

**Table 4:** Results from IDEXX PCR pathogen testing on fecal samples from six kennels.

	**Randomly selected kennel**						
**Pathogen**	**110**	**119**	**121**	**130**	**106**	**104**	**Percent Positive**
Canine Distemper Virus	+	+	+	+	+	+	100
Canine Enteric Coronavirus	+	+	–	+	–	–	50
*C. perf* alpha toxin gene	+	–	+	–	–	–	33.3
*C. perf* CPnetEF Toxin Gene	+	–	+	–	–	–	33.3
*Campylobacter jejuni*	–	–	+	–	–	–	16.7
Giardia sp.	–	–	–	+	–	–	16.7
*Campylobacter coli*	–	–	–	–	–	–	0
*C. difficile* toxin A/B	–	–	–	–	–	–	0
*Circovirus*	–	–	–	–	–	–	0
*C. perf* eneterotoxin gene	–	–	–	–	–	–	0
*Cryptosporidium*	–	–	–	–	–	–	0
Canine Parvovirus 2	–	–	–	–	–	–	0
*Salmonella*	–	–	–	–	–	–	0

### Vaccination Outcomes

**Figure 1:**
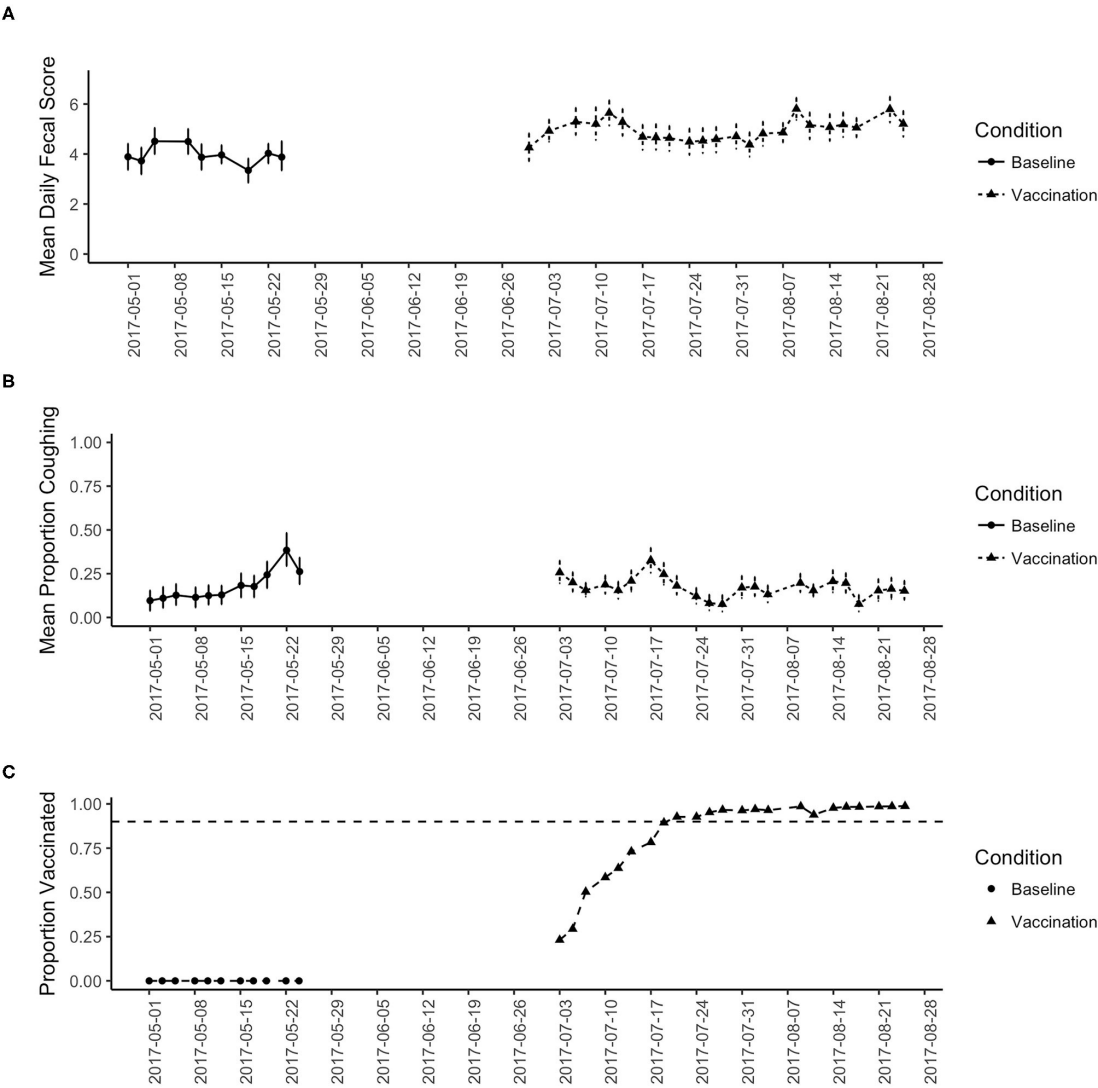
**(A)** shows the change in the mean fecal score across the study period for the baseline and vaccination period. Date shows calendar date. **(B)** shows the change in the proportion of dogs coughing across the study period for the baseline and vaccination period. **(C)** Shows the proportion of dogs vaccinated across the study period. Reaching the 90% criterion was slow as vaccination was implemented on intake, then stabilized near 100% for the remainder of the study.

Figure 1 shows the overall results for this study. Figure 1A shows the change in mean daily fecal score across the study period. During baseline (no vaccination), the fecal score averaged around a 4 (soggy stool that is still in a distinct log form). During the study period, however, the fecal score showed a slight increase indicating looser stools.

Figure 1B shows the change in daily proportion of dogs coughing and Figure 1C shows the proportion of dogs in the shelter that were vaccinated. During baseline, zero dogs were provided vaccination (per shelter protocol). From Figure 1B we see an increasing trend in the proportion of dogs coughing, which likely reflects the increase in seasonal trends. Figure 1B shows that at the beginning of the vaccination phase, the proportion coughing was as high as baseline, leveling off around 25% of the dogs coughing. However, as the proportion of the dogs vaccinated increases (Figure 1C), the proportion of dogs coughing decreases.

**Figure 2:**
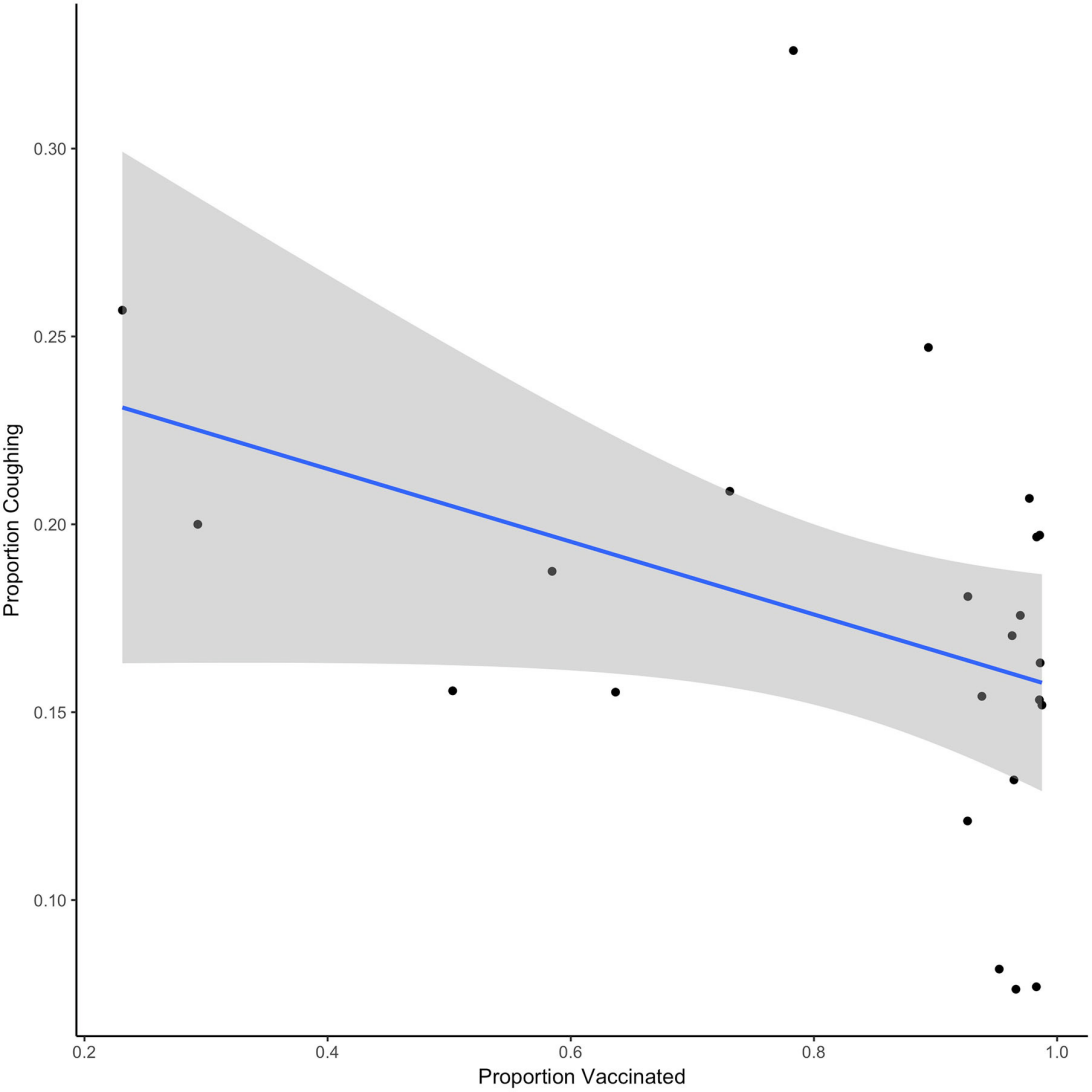
Shows the relationship between the proportion vaccinated and the proportion of dogs coughing for each day of the vaccination phase. Line shows the best fit linear regression and shaded region shows the 95% CI.

To evaluate this relationship statistically, we focused on the variability in the proportion of dogs vaccinated during the vaccination phase and the proportion of dogs coughing to identify a potential relationship. We focused this analysis on the vaccination phase only, given the time difference between the baseline data and potential seasonal effects. Given that by implementing vaccination on intake, there is a significant delay between the start of the vaccination period and reaching a total immunization status, we investigated this variability in Figure 2. Figure 2 shows the daily data from the vaccination phase in which we recorded that day's proportion of animals vaccinated and proportion of animals coughing. Although we quickly reached a high level of vaccination, Figure 2 shows that as the proportion of vaccinated dogs increased, the proportion of dogs coughing generally decreased. A linear regression model indicates that there was an overall trend association between the proportion of dogs vaccinated and the proportion of dogs coughing (estimate = 0.097, SE = 0.051, *t* = −1.899, *p* = 0.07).

**Figure 3:**
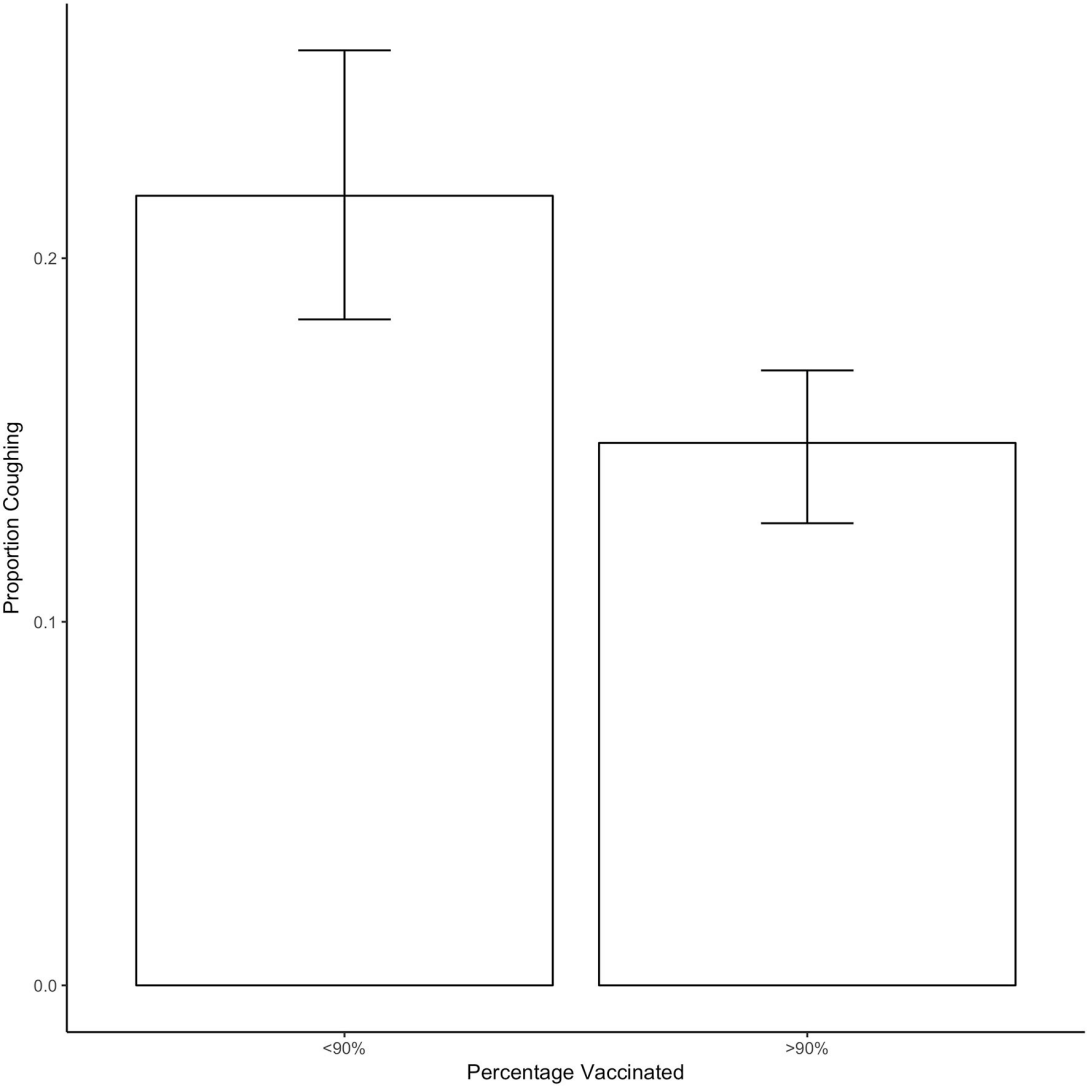
Shows the proportion of dogs coughing as a function of whether <90 or 90% or more of dogs in the shelter were vaccinated. Bars show the mean and error bars show the 95% CI.

Figure 3 shows the proportion of dogs coughing when vaccination rates equaled or exceeded 90% or when they were <90%. On average, there was a significant decrease of 7% of the proportion of dogs coughing when vaccination was at least at 90% compared to when it was <90% (estimate = −0.07, SE = 0.021, *t* = −3.193, *p* = 0.004).

**Figure 4:**
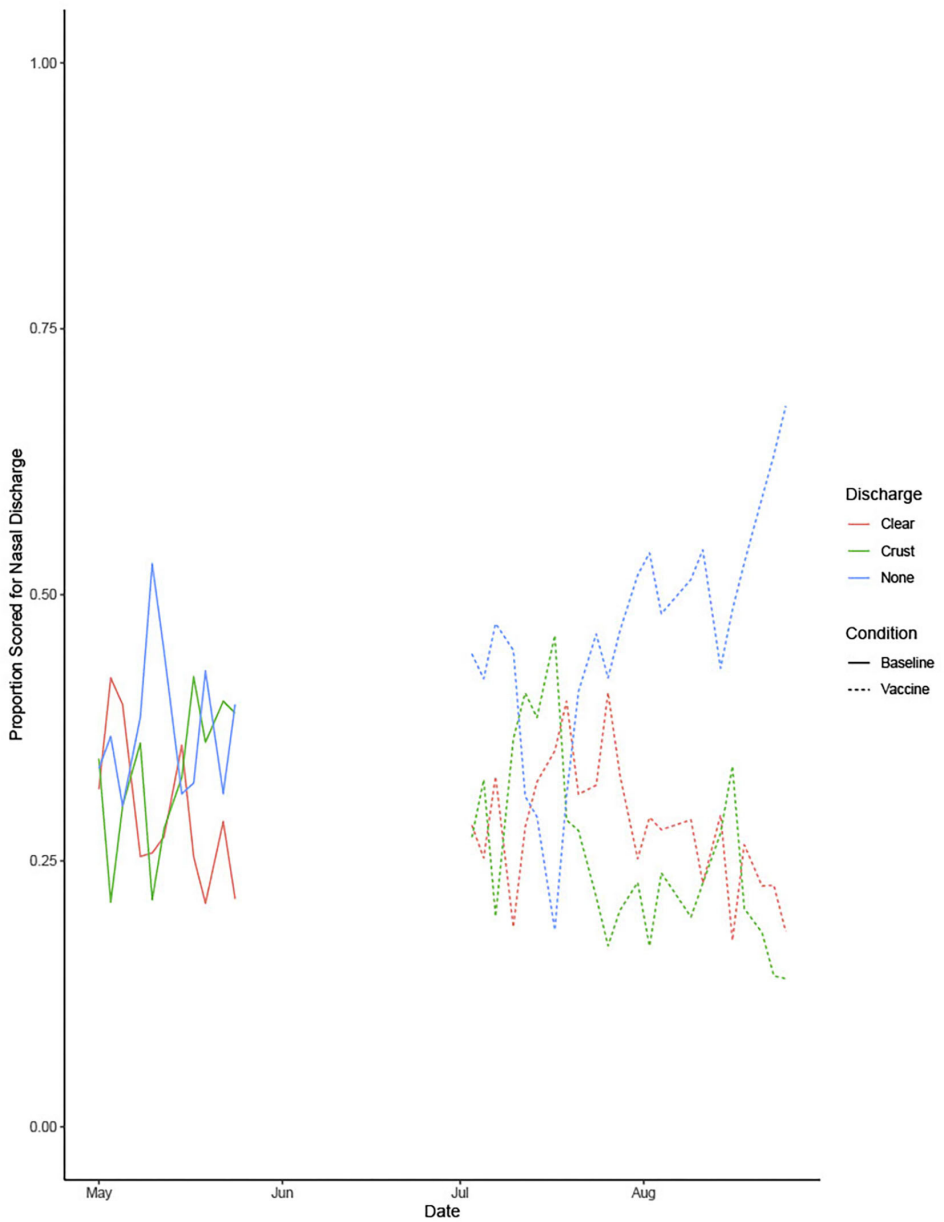
Shows the proportion of dogs with each nasal discharge type during the baseline and vaccine conditions.

Figure 4 shows the proportion of dogs with each nasal discharge type in both the baseline and vaccination periods. The proportion of dogs with clear or other discharge decreased during the vaccination period, whereas the proportion of dogs with no nasal discharge increased.

**Figure 5:**
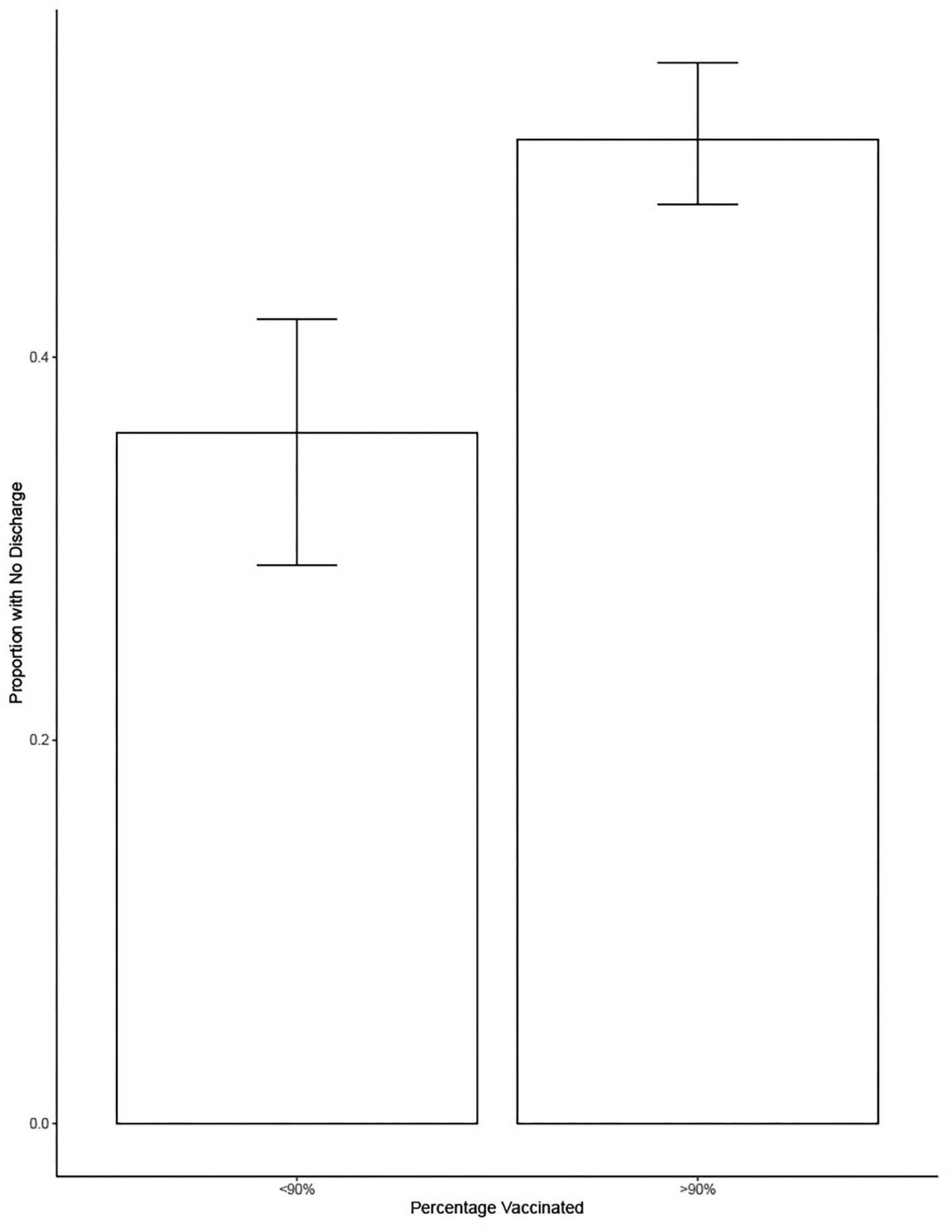
Shows the proportion of dogs with no nasal discharge as a function of whether <90 or 90% or more of dogs in the shelter were vaccinated. Bars show the mean and error bars show the 95% CI.

Figure 5 shows the proportion of dogs with no nasal discharge when vaccination rates equaled or exceeded 90% or when they were <90%. On average, there was a significant increase of 15% of the proportion of dogs with no nasal discharge when vaccination is over 90% compared to when it was under 90% (estimate = 0.15, *se* = 0.03733, *t* = 4.097, *p* = 0.0005).

## Discussion

This study was designed for a multitude of reasons. The objectives ranged from a highly localized, applied aim of establishing a vaccination-at-intake protocol at an open-admission municipal shelter to a novel question of whether it is possible to reduce clinical signs of CIRD in a shelter population with intake vaccinations even during an outbreak.

All of the dogs swabbed tested positive for at least one pathogen, supporting previous research that CIRD is common in the shelter environment (12, 34). This exceeds previous findings in asymptomatic United States shelter dogs (35) and Brazilian shelter dogs housed in sub-optimal conditions (36) which found that 47.7 and 78% respectively were positive for CIRD pathogens. Not only did 100% of the dogs test positive for at least one pathogen, but 70% tested positive for multiple pathogens. Co-infections have been previously found to increase disease severity and increase mortality rates (37–40).

We found 100% of the kennels and 80% of the individual dogs tested were positive for CDV. Whereas, other cities within the United States have recently reported an increase in CDV cases, the number of positives was still below 30% (41). A recent metanalysis on CDV suggested that the average positivity rate for CDV is 34% (42). The positivity rate in the Lubbock shelter was over twice the average previously reported, suggesting they were experiencing a concerning outbreak. Given that distemper has a mortality rate of ~50%, this is of particular concern (16). Due to restrictions placed on the research team by the shelter, it was not possible to accurately quantify the mortality rate, but it was likely to be a substantial number of dogs.

Under many circumstances and for many diseases, it is suggested that vaccination must reach a threshold (i.e., herd immunity) to have significant effects on the population. The vaccination threshold varies across diseases and populations (43). This study evaluated the effect of a 90% vaccination rate and found a significant impact. Due to the correlational nature of the study, we only evaluated this high-level criterion as the data collection for the project was secondary to implementation of vaccination and were not able to evaluate the effects of multiple vaccination rates. Future research should examine the threshold longitudinally across numerous shelters to see if the 90% remains effective. A longitudinal study may provide insight into waning vaccine-induced immunity and a possible need for boosters. While the booster vaccination protocol is well-established in puppies (44), there is less information on re-vaccination in animal shelters. Due to the increased susceptibility to disease in animals housed in the shelter environment, and the limited budgets of most municipal shelters, it is vital that researchers establish a vaccination and booster schedule that maximizes immunity with minimal cost.

We unexpectedly saw an increase in fecal scores during the vaccination period. While vaccinations can cause diarrhea, it is not common in the Nobivac^®^ Canine 1-DAPPv 5 Way nor Nobivac^®^ Intra-Trac^®^ 3 vaccine (45, 46). The increase in fecal score was likely due to a seasonal effect. The average daily temperature during baseline (May) was 21.1 C. In contrast, the average temperature during the experimental phase ranged from 27.2 C (July) to 24.4 C (August). A longitudinal study on gastrointestinal issues in dogs found the lowest incidence of vomiting and diarrhea in May and the highest incidence of vomiting and diarrhea in August and September (47).

## Limitations

Though we believe the reported results are illuminating and useful, there were limitations to our methodology that should be considered. The first limitation was that, due to budgetary constraints, pathogens were not measured following the implementation of the vaccine procedure. While we did see a decrease in *clinical signs* associated with CIRD, we do not have the PCR results to support the claim that the vaccination procedure decreased the prevalence of CIRD. The results would certainly be more robust had we included pre and post pathogen testing to get an accurate picture of the change in pathogen load in the population.

Another limitation to our methodology was that we could not control for seasonal effects; changes in temperature could have impacted our results. Previous research has suggested an increase in CIRD (48) and more specifically, CDV (49–51) during the cold months. This would suggest that seasonal changes may have influenced our decrease in the prevalence of coughing. However, Maboni et al. (38) found an increase in CIRD samples sent to be tested in the warm months (April through October) and no difference in the detection of pathogens between warm and cold months. Wyllie et al. (52) saw an increase in positive cases of CDV during the summer. It is important to note that this study included both canines and ferrets and did not separate out the two in seasonal data analysis (52). While it is possible that seasonal changes had an influence on the decrease in coughing, it is unlikely that seasonal changes accounted for the entirety of the change.

Finally, the researchers (AA, KB, AP) who collected the health data also designed the study and administered the vaccines, and thus were not blind to the hypotheses. This was due to the scope of the study which required researcher presence during all shelter operating hours for several months, as well as the established working relationships the researchers had forged with the animal shelter. We did, however, document acceptable inter-rater reliability, and the swabs collected demonstrated pathogen presence for dogs deemed ill by researchers' visual inspection.

Despite these limitations, we expect our findings to be useful for veterinary medicine generally, but particularly so for the shelter medicine community, and even more so for organizations that do not have mandatory intake vaccination protocol. There are several reasons that dogs were not vaccinated on intake at Lubbock Animal Services prior to this study. The primary reasons were previous misinformation on the importance and efficacy of vaccines and lack of funding. For these reasons, intake vaccinations were dismissed. Instead, vaccines were given only to dogs that staff planned to move to the adoption floor in a different wing of the shelter. While there is no published data on worldwide animal shelter vaccination protocols, it is unlikely that the situation at this municipal shelter is unique. There are likely other shelters, both within the United States and abroad, that face the same challenges and circumstances that were present during our baseline phase. These findings will be useful for other shelters that struggle with disease control and find themselves in similar dire situations.

## Conclusion

This study suggested that (1) intake vaccinations decrease proportion of dogs exhibiting signs of CIRD even during an outbreak and (2) the prevalence of coughing and nasal discharge significantly decreased when 90% of the population is vaccinated, possibly representing the vaccine threshold for CIRD. This study provides strong support for the importance of intake vaccinations in the animal shelter environment.

## Data Availability Statement

The raw data supporting the conclusions of this article will be made available by the authors, without undue reservation.

## Ethics Statement

All procedures were approved by Texas Tech University IACUC (protocols 17058-06 and 17060-06). At the time of this study, the conditions at the local shelter were substandard and vaccinations were given sparingly and at random. This study was conceived out of desperation, in an attempt to establish the efficacy of vaccinations on intake, even amidst an epidemic. We used the data from this study to demonstrate the importance of vaccinations on intake to the city of Lubbock. Since our findings were presented, the staff has continued to vaccinate on intake, there is a new director, a veterinarian is on staff, and distemper is no longer rampant in the shelter.

## Author Contributions

AP conceived the idea for this study. AP, AA, and KB devised the methods and collected the data. AA and AP secured funding for its success. NH analyzed the data and prepared the results and figures. AA wrote the first draft of this manuscripts. All authors contributed to the interpretation of the results and contributed to the writing and editing.

## Conflict of Interest

The authors declare that the research was conducted in the absence of any commercial or financial relationships that could be construed as a potential conflict of interest.
